# Palmatine Protects against Cerebral Ischemia/Reperfusion Injury by Activation of the AMPK/Nrf2 Pathway

**DOI:** 10.1155/2021/6660193

**Published:** 2021-03-11

**Authors:** Chaoliang Tang, Junmou Hong, Chengyun Hu, Chunxia Huang, Jie Gao, Jun Huang, Di Wang, Qingtian Geng, Yongfei Dong

**Affiliations:** ^1^Department of Anesthesiology, The First Affiliated Hospital of USTC, Division of Life Sciences and Medicine, University of Science and Technology of China, Hefei, Anhui 230001, China; ^2^Department of Vascular Surgery, Zhongshan Hospital, Xiamen University, Xiamen, Fujian 361004, China; ^3^Department of Anesthesiology, The Second Affiliated Hospital of Anhui Medical University, Hefei, Anhui 230601, China; ^4^Department of Anesthesiology, The First Affiliated Hospital of Anhui Medical University, Hefei, Anhui 230022, China; ^5^Department of Anesthesiology, The People's Hospital of Chizhou, Chizhou, Anhui 247000, China; ^6^Department of Neurosurgery, The First Affiliated Hospital of USTC, Division of Life Sciences and Medicine, University of Science and Technology of China, Hefei, Anhui 230001, China

## Abstract

Palmatine (PAL), a natural isoquinoline alkaloid, possesses extensive biological and pharmaceutical activities, including antioxidative stress, anti-inflammatory, antitumor, neuroprotective, and gastroprotective activities. However, it is unknown whether PAL has a protective effect against ischemic stroke and cerebral ischemia/reperfusion (I/R) injury. In the present study, a transient middle cerebral artery occlusion (MCAO) mouse model was used to mimic ischemic stroke and cerebral I/R injury in mice. Our study demonstrated that PAL treatment ameliorated cerebral I/R injury by decreasing infarct volume, neurological scores, and brain water content. PAL administration attenuated oxidative stress, the inflammatory response, and neuronal apoptosis in mice after cerebral I/R injury. In addition, PAL treatment also decreases hypoxia and reperfusion- (H/R-) induced neuronal injury by reducing oxidative stress, the inflammatory response, and neuronal apoptosis. Moreover, the neuroprotective effects of PAL were associated with the activation of the AMP-activated protein kinase (AMPK)/nuclear factor E2-related factor 2 (Nrf2) pathway, and Nrf2 knockdown offsets PAL-mediated antioxidative stress and anti-inflammatory effects. Therefore, our results suggest that PAL may be a novel treatment strategy for ischemic stroke and cerebral I/R injury.

## 1. Introduction

Stroke, an acute cerebrovascular disease, is an important contributor to mortality and permanent disability worldwide [1, 2]. Epidemiological data showed that ischemic strokes account for approximately 87% of these incidences [3, 4]. Currently, early restoration of the blood supply is considered the main treatment strategy for acute ischemic stroke. However, reperfusion processes after ischemic attack may further exacerbate brain damage, which is named cerebral ischemia/reperfusion (I/R) injury [5, 6]. It is increasingly understood that multiple pathophysiological processes, including oxidative stress, the inflammatory response, neuronal death, and apoptosis, play a pivotal role in the development of cerebral I/R injury [7–10]. Thus, treatments based on the above mechanisms are proposed as a promising strategy to attenuate the outcomes of stroke and cerebral I/R injury.

Palmatine (PAL) is a natural isoquinoline alkaloid extracted from *Coptidis rhizome* [11, 12]. A growing body of evidence indicates that PAL possesses extensive biological and pharmaceutical activities, including antioxidative stress, anti-inflammatory, antitumor, neuroprotective, and gastroprotective activities [11, 13, 14]. Lee et al. reported that PAL treatment inhibited the inflammation and apoptosis of hepatocytes during acute liver injury [15]. In addition, PAL alleviated ulcerative colitis-induced injury by preserving the integrity of intestinal barrier and mitigating colonic inflammation [16]. Kim et al. also reported that PAL attenuated myocardial I/R injury via suppressing oxidative stress and the inflammatory response [17]. However, it is unknown whether PAL has protective effect against ischemic stroke and cerebral I/R injury.

In the present study, we found that PAL exerts its neuroprotective effect by attenuating cerebral I/R-induced oxidative stress, neuroinflammation, and neuronal apoptosis. In addition, the protective function of PAL in cerebral I/R injury is involved in the activation of the AMP-activated protein kinase (AMPK)/nuclear factor E2-related factor 2 (Nrf2) signaling pathway. Therefore, our results indicate that PAL may be a novel treatment strategy for ischemic stroke and cerebral I/R injury.

## 2. Materials and Methods

### 2.1. Animal and Animal Experiments

Nine- to ten-week-old C57BL/6J mice (*n* = 120) housed in a barrier system with free access to food and water. All procedures were approved by the Animal Experimentation Ethics Committee of Anhui Medical University. The transient middle cerebral artery occlusion (MCAO) model in mice was performed by occluding the middle cerebral artery (MCA) according to our previous research [18]. The sham-operated mice underwent the same protocol, but without MCA ligation. One hour before cerebral ischemia, PAL (MedChemExpress LLC., USA) was dissolved in physiological saline solution (PBS) and was administered orally (50 or 100 mg/kg) to the animals. The 120 mice were randomly allocated into the following four groups (*n* = 30/group): sham group, MCAO group, PAL (50 mg/kg) group, and PAL (100 mg/kg) group.

### 2.2. Neurological Deficits Evaluation

After reperfusion for 24 h, neurological deficits were assessed according to the following scoring criteria [19]: 0, no neurological deficits; 1, unable to extend the contralateral forelimb; 2, circling to paretic side; 3, falling to the contralateral side; and 4, unable to engage in spontaneous activity.

### 2.3. Infarct Volume Measurement

Mice were euthanized using 2% pentobarbital sodium, and intact brains were rapidly collected after neurological deficit evaluation. The brains were cut into four coronal sections and incubated with 2% TTC solution. All images were collected and analyzed using ImageJ (NIH, USA), as described previously [20].

### 2.4. Brain Water Content

Brain water content was assessed using the wet/dry method according to our previous research [18]. Briefly, the brains were carefully removed and promptly weighed to measure the wet weight. Dry weight was evaluated after the brains were dried at 105°C for 24 h.

### 2.5. Cell Culture and Treatment

The PC12 cells were purchased from the China Centre for Type Culture Collection and cultured according to our previous research [18]. First, the PC12 cells were transferred to a hypoxic incubator with 95% N_2_/5% CO_2_ and placed in a CB-210 hypoxia workstation (BINDER, Germany) for 6 h. Next, the medium was replaced with fresh maintenance medium and recovered under normoxic conditions for 18 h. In addition, the cells were treated with PAL for 6 h before hypoxic treatment. To knockdown Nrf2, PC12 cells were transfected with si-Nrf2 (Invitrogen, USA) using Lipofectamine 2000 (Thermo Fisher Scientific, USA) according to the manufacturer's recommendation.

### 2.6. Oxidative Stress Measurement

8-Hydroxydeoxyguanosine (8-OHdG) staining was performed as previously described [21]. Briefly, the brain tissues were quickly isolated and sectioned at a thickness of 4 *μ*m. Then, the sections were incubated with 8-OHdG monoclonal antibody (Sigma, USA) and placed under a fluorescence microscope (Olympus, Japan). In addition, superoxide dismutase (SOD) and catalase (CAT) activities, and malondialdehyde (MDA) contents in the brain tissues and PC12 cells were measured according to the protocol recommended (Beyotime Biotechnology, China).

### 2.7. ELISA Analysis

The brain tissues were quickly isolated and prepared as homogenates in a homogenizer. The concentrations of interleukin- (IL-) 1*β*, IL-6, and tumor necrosis factor- (TNF-) *α* were detected using ELISA kits (R&D Systems, USA).

### 2.8. TUNEL Staining

TUNEL staining was performed to detect the extent of cell apoptosis as previously described [22]. The images were collected using an automatic fluorescence microscope and analyzed using ImageJ (NIH, USA).

### 2.9. Western Blot

Total proteins extracted from ischemic side cerebral cortex and PC12 cells were collected and fractionated on SDS-PAGE gels [23]. Then, the protein was incubated with primary antibodies against Bax (1 : 1000, Abcam, USA), Bcl-2 (1 : 1000, Abcam, USA), p-AMPK (1 : 1000, Abcam, USA), AMPK (1 : 1000, Abcam, USA), Nrf2 (1 : 1000, Cell Signaling Technology, USA), lamin B (1 : 1000, Abcam, USA), and *β*-actin (1 : 1000, Abcam, USA). After that, the membranes were incubated with the corresponding secondary antibodies (1 : 5000, Abcam, USA) and scanned using Odyssey imaging system (LI-COR, USA). The relative band intensity was normalized to that of *β*-actin or lamin B.

### 2.10. qRT-PCR

Total RNA was extracted from ischemic side cerebral cortex and PC12 cells according to the manufacturer's instructions [24]. Then, total RNA was reverse-transcribed, and amplification was quantified using SYBR Premix Ex Taq2. The mRNA expression level was normalized to the *β*-actin level. Primer sequences of qRT-PCR analysis are presented in Table 1.

### 2.11. Statistical Analysis

The population data are expressed as the means ± SD. One-way analysis of variance (ANOVA) tests were used to examine the statistical difference. *P* values below 0.05 were considered to be significant.

## 3. Results

### 3.1. PAL Protected Mice against Cerebral I/R Injury

**Figure 1:**
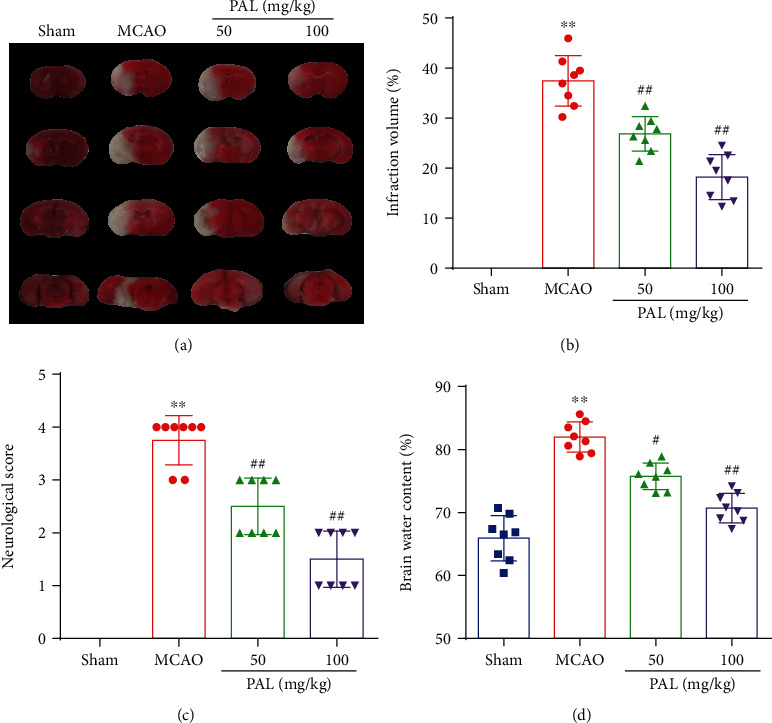
PAL protected mice against cerebral I/R injury. (a, b) Effects of PAL on infarct volume (*n* = 8). (c) Effects of PAL on neurological scores (*n* = 8). (d) Effects of PAL on brain water content (*n* = 8). ^∗∗^*P* < 0.01 vs. sham group; ^#^*P* < 0.05 and ^##^*P* < 0.01 vs. MCAO group. PAL: palmatine.

As shown in Figure 1, the MCAO model resulted in an increase in the infarct volume and neurological scores compared with those of the sham group. In addition, PAL treatment significantly decreased the infarct volume and neurological scores at 24 h after MCAO (Figures 1(a)–1(c)). As anticipated, PAL treatment also reduced brain water content in a dose-dependent manner (Figure 1(d)). Together, these findings strongly support that PAL plays a protective role in mouse cerebral I/R injury.

### 3.2. PAL Suppresses Oxidative Stress after Cerebral I/R Injury

**Figure 2:**
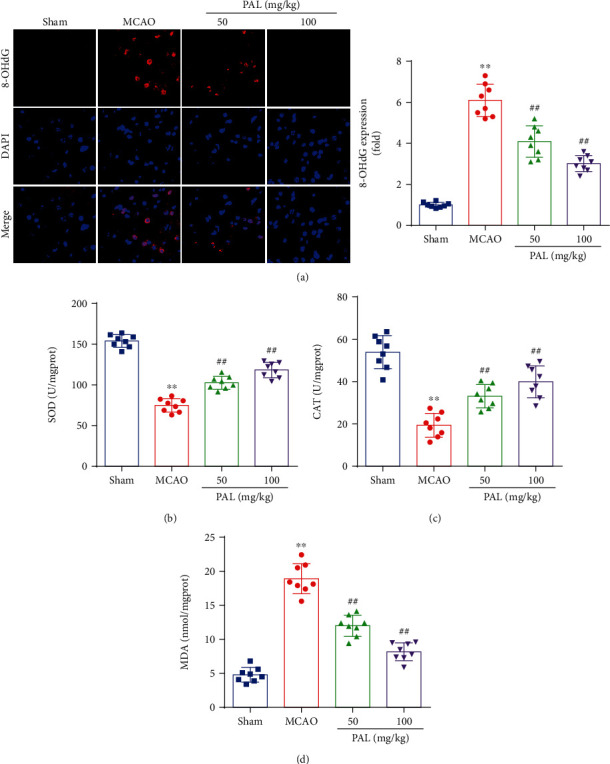
PAL suppresses oxidative stress after cerebral I/R injury. (a) 8-OHdG staining and quantitative analysis (*n* = 8; scale bar, 25 *μ*m). (b–d) Effects of PAL on SOD and CAT activities, and MDA contents (*n* = 8). ^∗∗^*P* < 0.01 vs. sham group; ^##^*P* < 0.01 vs. MCAO group. PAL: palmatine.

Oxidative stress has been proven to be a critical pathological process responsible for ischemic stroke and cerebral I/R injury [25]. We therefore investigated the effect of PAL on oxidative stress. Consistent with previous studies, 8-OHdG expression in the MCAO group was higher than that in the sham group, which was mitigated by PAL (Figure 2(a)). Moreover, a significant reduction in SOD and CAT activities and an increase in MDA contents were also observed in the MCAO group compared with the sham group (Figures 2(b)–2(d)). Interestingly, PAL administration significantly restored SOD and CAT activity, and reduced the MDA content, in the brain after MCAO (Figures 2(b)–2(d)).

### 3.3. PAL Reduces the Inflammatory Response after Cerebral I/R Injury

**Figure 3:**
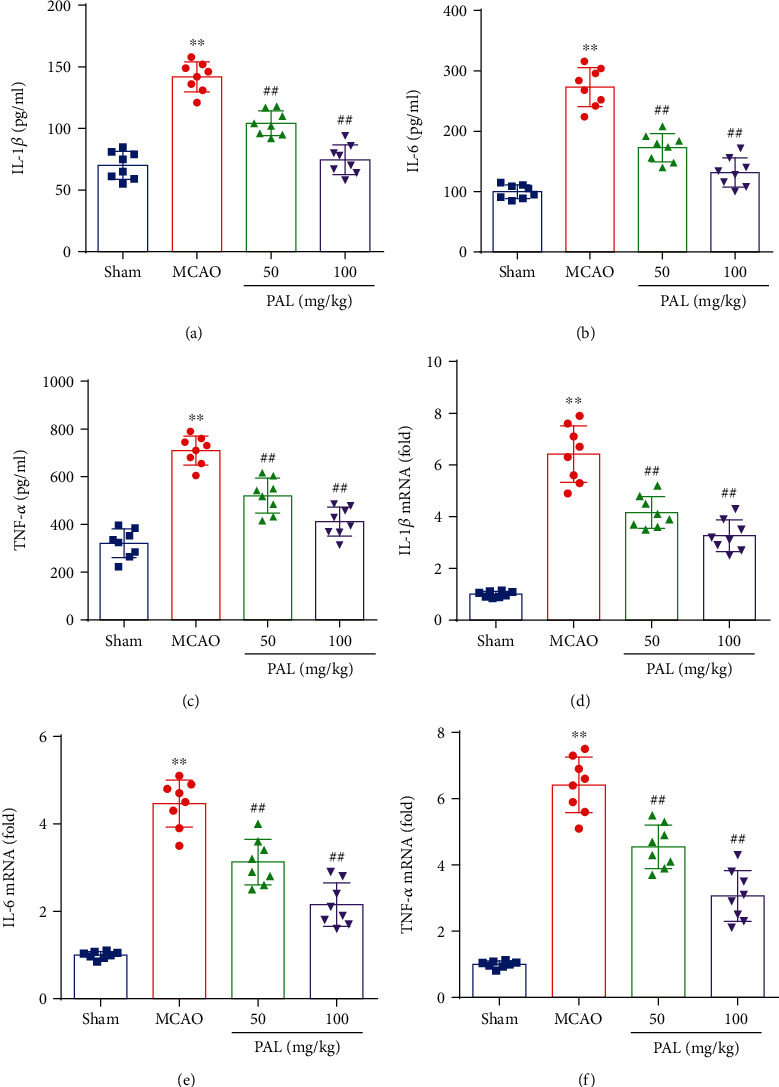
PAL reduces the inflammatory response after cerebral I/R injury. (a–c) The expression of IL-1*β*, IL-6, and TNF-*α* protein in the ischemic cerebral cortex (*n* = 8). (d–f) The relative mRNA levels of IL-1*β*, IL-6, and TNF-*α* in the ischemic cerebral cortex (*n* = 8). ^∗∗^*P* < 0.01 vs. sham group; ^##^*P* < 0.01 vs. MCAO group. PAL: palmatine.

Previous studies have found that the inflammatory response is an important mediator of ischemic stroke and cerebral I/R injury [26]. Thus, we evaluated the potential role of PAL in the inflammatory response after cerebral I/R injury. As shown in Figures 3(a)–3(c), the levels of inflammatory cytokines, including IL-1*β*, IL-6, and TNF-*α*, were significantly increased in the MCAO group compared with the sham group. In addition, PAL treatment prevented the increase in IL-1*β*, IL-6, and TNF-*α* in the brain after MCAO (Figures 3(a)–3(c)). RT-PCR results also showed that PAL treatment reduces the mRNA expression of these inflammatory cytokines (Figures 3(d)–3(f)), indicating that PAL treatment reduces the inflammatory response after cerebral I/R injury.

### 3.4. PAL Attenuates Neuronal Apoptosis after Cerebral I/R Injury

**Figure 4:**
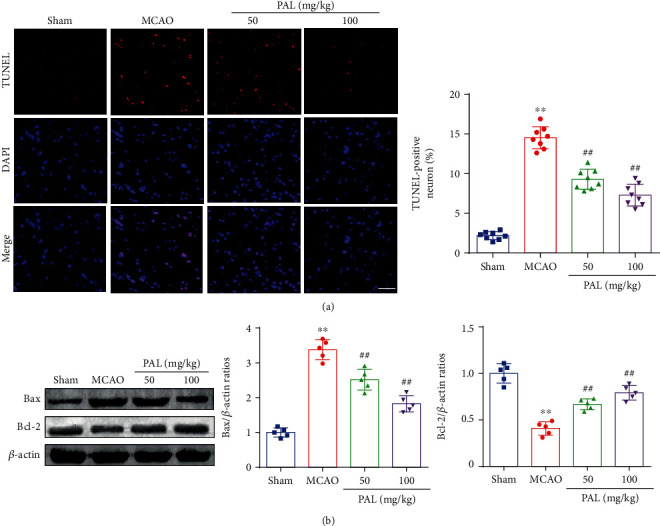
PAL attenuates neuronal apoptosis after cerebral I/R injury. (a) TUNEL staining and quantitative analysis (*n* = 8; scale bar, 50 *μ*m). (b) Effects of PAL on Bax and Bcl-2 expression (*n* = 5). ^∗∗^*P* < 0.01 vs. sham group; ^##^*P* < 0.01 vs. MCAO group. PAL: palmatine.

TUNEL staining results showed that neuronal apoptosis was increased in the MCAO group compared with the sham group but reduced in PAL-treated mice (Figure 4(a)). In addition, the expression of Bax in the MACO group was higher and the expression of Bcl-2 in the MACO group was lower than that in the sham group (Figure 4(b)). However, PAL administration significantly decreased Bax expression and upregulated Bcl-2 expression at 24 h after MCAO (Figure 4(b)).

### 3.5. PAL Reduces Oxidative Stress, the Inflammatory Response, and Neuronal Apoptosis In Vitro

**Figure 5:**
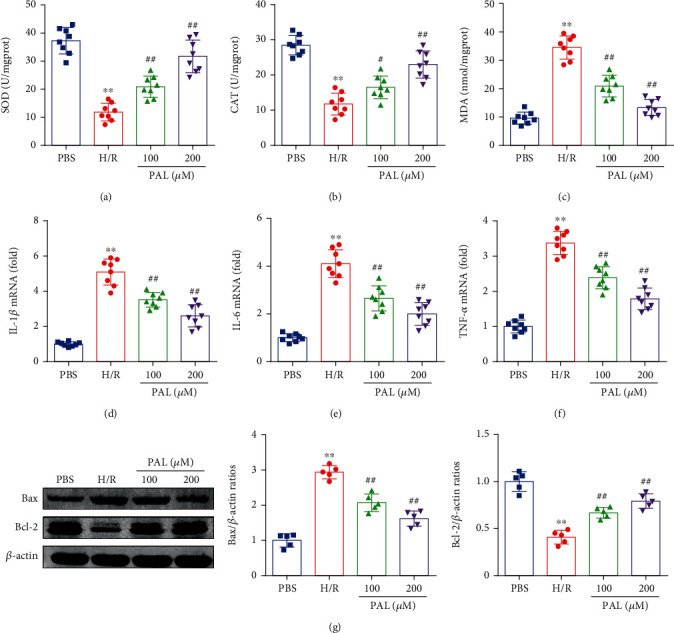
PAL reduces oxidative stress, the inflammatory response, and neuronal apoptosis in vitro. (a–c) Effects of PAL on SOD and CAT activities, and MDA contents (*n* = 8). (d–f) The relative mRNA levels of IL-1*β*, IL-6, and TNF-*α* (*n* = 8). (g) Effects of PAL on Bax and Bcl-2 expression (*n* = 5). ^∗∗^*P* < 0.01 vs. PBS group; ^##^*P* < 0.01 vs. H/R group. PAL: palmatine.

As shown in Figures 5(a)–5(c), a significant reduction in SOD and CAT activities and an increase in MDA contents were observed in the H/R group compared with the control group (Figures 5(a)–5(c)). In addition, PAL administration significantly restored SOD and CAT activity, and reduced the MDA content in PC12 cells following H/R (Figures 5(a)–5(c)). Consistent with the results of animal experiments, PAL administration also significantly decreased the mRNA expression of IL-1*β*, IL-6, and TNF-*α* in PC12 cells following H/R (Figures 5(d)–5(f)). In addition, the expression of Bax in the H/R group was higher, and the expression of Bcl-2 in the H/R group was lower than that in the PBS group (Figure 5(g)). However, PAL administration significantly decreased Bax expression and upregulated Bcl-2 expression in PC12 cells following H/R (Figure 5(g)).

### 3.6. PAL Activates the AMPK/Nrf2 Signaling Pathway

**Figure 6:**
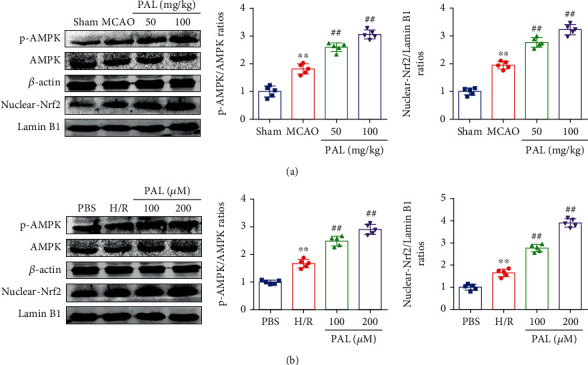
PAL activates the AMPK/Nrf2 signaling pathway. (a) Effects of PAL on p-AMPK, AMPK, and nuclear Nrf2 expression in the ischemic cerebral cortex (*n* = 5). (b) Effects of PAL on p-AMPK, AMPK, and nuclear Nrf2 expression in PC12 cells (*n* = 5). ^∗∗^*P* < 0.01 vs. sham or PBS group; ^##^*P* < 0.01 vs. MCAO or H/R group. PAL: palmatine.

Previous research has strongly suggested that the AMPK/Nrf2 signaling pathway plays an important role in cerebral I/R injury [27, 28]. Hence, we detected whether the neuroprotective function of PAL is associated with the AMPK/Nrf2 signaling pathway. The results revealed that the phosphorylation level of AMPK and the expression of nuclear Nrf2 were upregulated in the MCAO group compared with the sham group (Figure 6(a)). In addition, PAL administration further increased the phosphorylation level of AMPK and the expression of nuclear Nrf2 after cerebral I/R injury (Figure 6(a)). Consistent with the results of animal experiments, PAL administration also significantly increased the phosphorylation level of AMPK and the expression of nuclear Nrf2 in PC12 cells following H/R (Figure 6(b)).

### 3.7. Nrf2 Knockdown Abolishes the Neuroprotective Effects of PAL In Vitro

**Figure 7:**
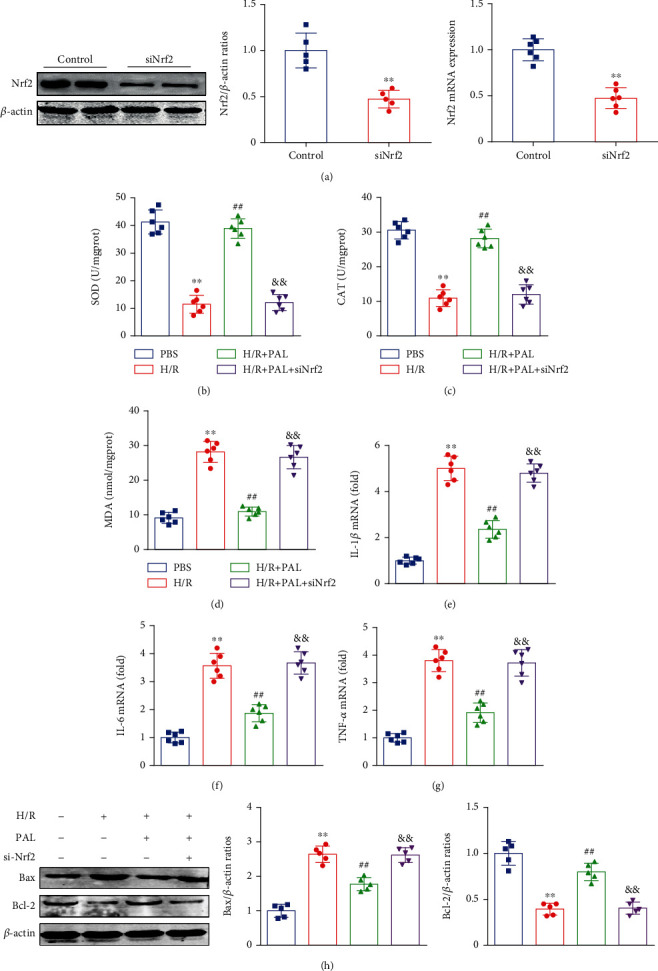
Nrf2 knockdown abolishes the neuroprotective effects of PAL in vitro. (a) Effects of Nrf2 expression after siRNA treatment (*n* = 5). (b–d) Effects of Nrf2 knockdown on SOD and CAT activities, and MDA contents (*n* = 6). (e–g) The relative mRNA levels of IL-1*β*, IL-6, and TNF-*α* (*n* = 6). (h) Effects of PAL on Bax and Bcl-2 expression (*n* = 5). ^∗∗^*P* < 0.01 vs. PBS group; ^##^*P* < 0.01 vs. H/R group; ^&&^*P* < 0.01 vs. H/R+PAL group. PAL: palmatine.

To further confirm the effect of the AMPK/Nrf2 signaling pathway in PAL-mediated neuroprotection, si-Nrf2 transfection was performed to knockdown Nrf2 in vitro. The results showed that Nrf2 knockdown abolished the PAL-mediated antioxidative stress effects, as evidenced by reduced SOD and CAT activities and increased MDA contents (Figures 7(a)–7(c)). In addition, Nrf2 knockdown offsets PAL-mediated anti-inflammatory and antineuronal apoptosis (Figures 7(d)–7(g)). The above results revealed that the AMPK/Nrf2 pathway plays a central role in PAL-mediated neuroprotective effects.

## 4. Discussion

In this study, our research revealed a neuroprotective function of PAL in mediating cerebral I/R injury. Our study demonstrated that PAL treatment ameliorated cerebral I/R injury by decreasing infarct volume, neurological scores, and brain water content. PAL administration attenuated oxidative stress, the inflammatory response, and neuronal apoptosis in mice after cerebral I/R injury. In addition, PAL treatment also decreased H/R-induced oxidative stress, the inflammatory response, and neuronal apoptosis in PC12 cells. Moreover, the neuroprotective function of PAL was associated with the activation of the AMPK/Nrf2 pathway, and Nrf2 knockdown offset PAL-mediated antioxidative stress and anti-inflammatory effects.

As a natural isoquinoline alkaloid, the neuroprotective effect of PAL has attracted the attention of many researchers [29, 30]. PAL was proven to treat Alzheimer's disease by reducing *β*-amyloid plaques and tau protein aggregation [29]. In addition, PAL exhibited antidepressant activity by decreasing nitrite and corticosterone levels and inhibiting monoamine oxidase-A activity [30]. However, the protective effect and the molecular mechanism of PAL in ischemic stroke and cerebral I/R injury are not well studied. In the present study, the MCAO mouse model was used to mimic ischemic stroke and cerebral I/R injury in vivo. The results showed that PAL treatment significantly decreased the infarct volume, neurological scores, and brain water content in mice at 24 h after MCAO, suggesting that PAL plays a protective role in ischemic stroke and cerebral I/R injury.

Reactive oxygen species (ROS) are crucial protagonists of oxidative stress, and causing neuronal injury and death [31, 32]. Multiple antioxidants and/or ROS scavengers have been shown to improve cerebral I/R injury and strongly support that suppressing oxidative stress is an attractive potential therapeutic target to counteract ischemic stroke and cerebral I/R injury [33, 34]. Previous research has shown that PAL possesses extensive biological and pharmaceutical activities [11, 29]. Thus, we investigated whether PAL affects oxidative stress after cerebral I/R injury.

In our study, 8-OHdG staining was performed to assess oxidative DNA damage and the results showed that 8-OHdG expression in the MCAO group was higher than that in the sham group, which was mitigated by PAL. Thus, it can be speculated that PAL could decrease oxidative DNA damage during cerebral I/R injury. SOD catalyzes the conversion of superoxide anion radicals to hydrogen peroxide, and the latter is further reduced into molecular oxygen and water by CAT [35, 36]. As a product of lipid peroxidation, MDA has been used to assess free radical levels in cerebral I/R injury [37]. Consistent with previous studies, significant reductions in SOD and CAT activities and increases in MDA contents were also observed after cerebral I/R injury. We further found that PAL significantly increased SOD and CAT activities, and reduced the MDA content in I/R mouse brain tissues and PC12 cells following H/R. These results directly reflect that PAL attenuates cerebral I/R injury by suppressing oxidative stress.

Intensive research has revealed that the inflammatory response is closely related to oxidative stress, and aggravating neuronal damage [38, 39]. In addition, proinflammatory cytokines, including IL-1, IL-6, IL-17, and TNF-*α*, have been detected in the brain of ischemic stroke patients and the infarction area of animal models [40, 41]. Previous studies have suggested that PAL has an anti-inflammatory effect in multiple diseases [11]. Thus, we evaluated the potential role of PAL in the inflammatory response after cerebral I/R injury. The results showed that PAL administration suppressed cerebral I/R-induced neuroinflammation by decreasing the expression of inflammatory cytokines, including IL-1*β*, IL-6, and TNF-*α*. Consistent with the results of animal experiments, PAL administration also significantly decreased the mRNA expression of IL-1*β*, IL-6, and TNF-*α* in PC12 cells following H/R. In addition, we further demonstrated the potential molecular mechanisms by which PAL exerts neuroprotective effects in cerebral I/R injury.

As a serine/threonine protein kinase, AMPK was proven to participate in the regulation of cellular stress and energy homeostasis and is considered as an important regulatory factor of oxidative stress and inflammation [27, 42]. Many results indicate that AMPK is activated after cerebral I/R injury and protects neurons from oxidative damage and inflammation [27, 43]. In addition, the neuroprotective potential of AMPK is closely related to the activation of Nrf2 signaling [28, 44]. In the current experimental protocol, we investigated whether the protective function of PAL is associated with the AMPK/Nrf2 signaling pathway. We found that PAL treatment significantly upregulated the phosphorylation of AMPK and nuclear Nrf2 expression after cerebral I/R injury. In addition, Nrf2 knockdown abolished the PAL-mediated antioxidative stress anti-inflammatory effects, indicating that the AMPK/Nrf2 pathway plays a central role in PAL-mediated neuroprotective effects.

**Figure 8:**
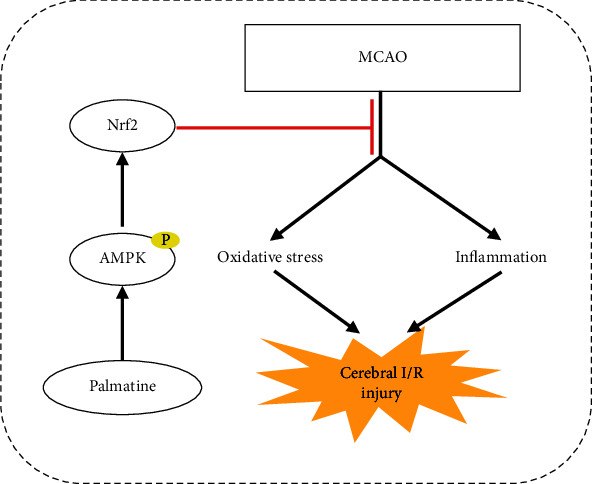
Palmatine protects against cerebral ischemia/reperfusion injury by activation of the AMPK/Nrf2 pathway.

In conclusion, PAL decreases oxidative stress, the inflammatory response, and neuronal apoptosis after cerebral I/R injury via activation of the AMPK/Nrf2 pathway (Figure 8). Our data indicate that PAL may be a novel therapeutic approach for ischemic stroke and cerebral I/R injury.

## Figures and Tables

**Table 1:** Primer sequences for RT-PCR assays.

Gene	Species	Sequence (5′-3′)	
IL-1*β*	Mouse	Forward	GGGCCTCAAAGGAAAGAATC
		Reverse	TACCAGTTGGGGAACTCTGC
IL-1*β*	Rat	Forward	GTGCTGTCTGACCCATGTGA
		Reverse	CACAGGGATTTTGTCGTTGCT
IL-6	Mouse	Forward	AGTTGCCTTCTTGGGACTGA
		Reverse	TCCACGATTTCCCAGAGAAC
IL-6	Rat	Forward	GTTGCCTTCTTGGGACTGATG
		Reverse	ATACTGGTCTGTTGTGGGTGGT
TNF-*α*	Mouse	Forward	CCCAGGGACCTCTCTCTAATC
		Reverse	ATGGGCTACAGGCTTGTCACT
TNF-*α*	Rat	Forward	CTACTCCCAGGTTCTCTTCAA
		Reverse	GCTGACTTTCTCCTGGTATGA
*β*-Actin	Mouse	Forward	TATTGGCAACGAGCGGTTCC
		Reverse	GGCATAGAGGTCTTTACGGATGT
*β*-Actin	Rat	Forward	CAAGAAGGTGGTGAAGCAG
		Reverse	AAAGGTGGAAGAATGGGAG

## Data Availability

The datasets generated and/or analyzed during the current study are available from the corresponding author on reasonable request in compliance with ethical standards.
